# Associations of passerine birds, rabbits, and ticks with *Borrelia miyamotoi and Borrelia andersonii* in Michigan, U.S.A.

**DOI:** 10.1186/1756-3305-5-231

**Published:** 2012-10-11

**Authors:** Sarah A Hamer, Graham J Hickling, Rich Keith, Jennifer L Sidge, Edward D Walker, Jean I Tsao

**Affiliations:** 1 Department of Fisheries and Wildlife, Michigan State University, East Lansing, MI 48823, USA; 2 Veterinary Integrative Bioscience Department, Texas A&M University, College Station, TX 77843, USA; 3 Center for Wildlife Health, University of Tennessee Institute of Agriculture, Knoxville, TN 37996, USA; 4 Kalamazoo Valley Bird Observatory, Kalamazoo Nature Center, Kalamazoo, MI 49009, USA; 5 Department of Microbiology and Molecular Genetics, Michigan State University, East Lansing, MI 48823, USA

**Keywords:** Ticks, *Borrelia miyamotoi*, *Borrelia andersonii*, *Ixodes*, Wild birds, Eastern cottontail rabbit, Relapsing fever, Lyme disease

## Abstract

**Background:**

Wild birds contribute to maintenance and dissemination of vectors and microbes, including those that impact human, domestic animal, and wildlife health. Here we elucidate roles of wild passerine birds, eastern cottontail rabbits (*Sylvilagus floridanus*), and *Ixodes dentatus* ticks in enzootic cycles of two spirochetes, *Borrelia miyamotoi* and *B. andersonii* in a region of Michigan where the zoonotic pathogen *B. burgdorferi* co-circulates.

**Methods:**

Over a four-year period, wild birds (n = 19,631) and rabbits (n = 20) were inspected for tick presence and ear tissue was obtained from rabbits. Samples were tested for *Borrelia* spirochetes using nested PCR of the 16S-23S rRNA intergenic spacer region (IGS) and bidirectional DNA sequencing. Natural xenodiagnosis was used to implicate wildlife reservoirs.

**Results:**

*Ixodes dentatus,* a tick that specializes on birds and rabbits and rarely bites humans, was the most common tick found, comprising 86.5% of the 12,432 ticks collected in the study. The relapsing fever group spirochete *B. miyamotoi* was documented for the first time in ticks removed from wild birds (0.7% minimum infection prevalence; MIP, in *I. dentatus*), and included two IGS strains. The majority of *B. miyamotoi*-positive ticks were removed from Northern Cardinals (*Cardinalis cardinalis*). *Borrelia andersonii* infected ticks removed from birds (1.6% MIP), ticks removed from rabbits (5.3% MIP), and rabbit ear biopsies (5%) comprised twelve novel IGS strains. Six species of wild birds were implicated as reservoirs for *B. andersonii.* Frequency of *I. dentatus* larval and nymphal co-feeding on birds was ten times greater than expected by chance. The relatively well-studied ecology of *I. scapularis* and the Lyme disease pathogen provides a context for understanding how the phenology of bird ticks may impact *B. miyamotoi* and *B. andersonii* prevalence and host associations.

**Conclusions:**

Given the current invasion of *I. scapularis*, a human biting species that serves as a bridge vector for *Borrelia* spirochetes, human exposure to *B. miyamotoi* and *B. andersonii* in this region may increase. The presence of these spirochetes underscores the ecological complexity within which *Borrelia* organisms are maintained and the need for diagnostic tests to differentiate among these organisms.

## Background

When considering the ecology of tick-borne diseases, it is becoming increasingly clear that wild birds maintain and move ticks and pathogens by serving as blood meal hosts and pathogen reservoirs. Birds have been implicated as reservoirs for several spirochetes within the genus *Borrelia* worldwide [1-5] and as vehicles for the long-distance dispersal of *Borrelia* spirochetes and ticks through their migratory movements [5-10]. For example, the phylogeographic structure of populations of three *Borrelia* species is congruent with vagility of vertebrate hosts, such that strains of the bird-associated *B. garinii* and *B. valaisiana* are spatially-mixed across countries, whereas strains of the rodent-associated *B. afzelii* are comparatively more differentiated geographically [11].

*Borrelia* spirochetes comprise three distinct species groups: (*i*) the Lyme borreliosis group, transmitted by hard ticks, which includes the agents of human Lyme disease such as *B. burgdorferi*, *B. afzelii*, and *B. garinii* as well as species not known to be pathogenic such as *B. andersonii*; (*ii*) the relapsing fever group, largely transmitted by soft ticks, which includes agents of human relapsing fever such as *B. duttonii* and *B. hermsii*; and (*iii*) a group that is most similar by molecular phylogenetic analysis to the relapsing fever spirochetes, but which are associated with hard tick vectors, including *B. theileri*, *B. lonestari*, and *B. miyamotoi*[12].

*Borrelia miyamotoi* is a relapsing fever group spirochete that was originally described in *I. persulcatus* in Japan [13] and later in *I. scapularis* in North America [14]. In North America, *B. miyamotoi* has been detected in the white-footed mouse *Peromyscus leucopus*[12,15,16] and wild turkey *Meleagris gallopavo*[17]. Given the apparent cosmopolitan association of *B. miyamotoi* within populations of human-biting *Ixodes* spp. ticks throughout North America and Europe, it is likely that humans are regularly exposed, albeit the *B. miyamotoi* infection prevalence (1.7-3.4% in adults and nymphs) is typically an order of magnitude less than that of *B. burgdorferi* infection prevalence in the same tick populations [12,18-22]. *B. miyamotoi* has recently been associated with relapsing fever and Lyme disease-like symptoms in humans in Russia [23], and has been found in ticks removed from humans in other countries [20]. Due to diagnostic testing specific for Lyme borreliosis group spirochetes, undetected human infection with *B. miyamotoi* in cases of Lyme disease-like illness is a possibility in North America and Europe.

*Borrelia andersonii* is a Lyme borreliosis group spirochete that was designated as a new species in 1995 [24] subsequent to its initial classification as an antigenic variant of *B. burgdorferi*[25,26]. *Borrelia andersonii* has not been implicated in human disease, which may reflect its association with *I. dentatus* - a tick that feeds almost exclusively on birds and rabbits [27]. Although direct human-biting by *I. dentatus* is rare, it has been documented several times across the *I. dentatus* range [28-34], and *B. andersonii* has been detected in an *I. dentatus* tick removed from a human in Connecticut [33]. Furthermore, *B. andersonii* was detected in a questing *I. scapularis* nymph [35] and in ear tissue of a white-footed mouse [16]; these observations suggest that *I. scapularis* may serve as a bridge vector of this spirochete to humans.

We recently identified a focal cryptic cycle of *B. burgdorferi* transmission maintained by several species of wild birds, eastern cottontail rabbits, and *Ixodes dentatus* ticks- a species that feeds almost exclusively on birds and rabbits [36] and postulated that on-going range expansions of the bridge vector *I. scapularis*[16] into such zones of cryptic pathogen maintenance could result in increased risk of human exposure. In this study, our objectives were to determine prevalence, host and vector associations, and phenology of other *Borrelia* species of potential public health importance, including *B. miyamotoi* and *B. andersonii,* that may be co-circulating within avian host and cryptic vector populations. We hypothesized that the phenology of bird-associated ticks would help to explain the observed patterns of pathogen prevalence.

## Methods

### Wildlife trapping

**Figure 1:**
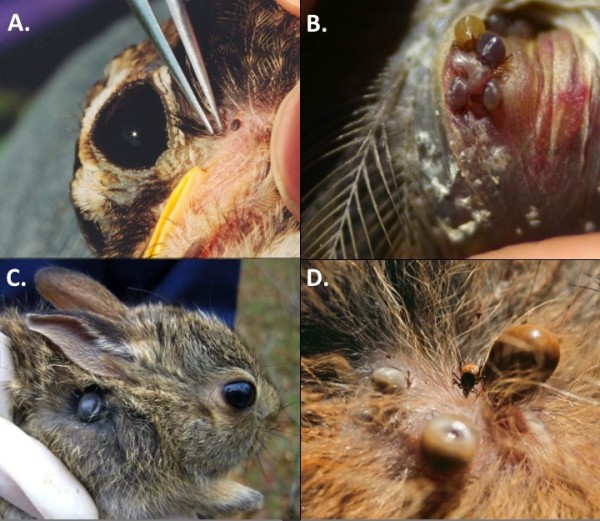
**Field investigations of wild birds and eastern cottontails for the presence of ticks.A**) American Robin harbors a single nymphal tick (near the tip of forceps); **B**) multiple engorged larval ticks are present beneath the auricular feathers and within the skin of the ear canal of a White-throated Sparrow; **C**) juvenile eastern cottontail harbors an engorged adult *I. dentatus* near the scruff; **D**) adult ticks of differing engorgement status are present on an eastern cottontail. Photo credits: Gabriel Hamer and Graham Hickling.

From 2004–2007, wild birds and eastern cottontail rabbits were captured and released alive using traps as described previously [36] at the Kalamazoo Valley Bird Observatory in Vicksburg, southwestern MI. This site is 90 km from the nearest coastal forests along Lake Michigan where *I. scapularis* ticks recently invaded and established populations [16,37]. Birds were captured 3 days per week during the breeding season (16 May- 31 August) and 5 days per week during the fall migratory season (1 September- 13 November). Birds and rabbits were examined for ticks (Figure 1), which were removed and preserved in 70% ethanol. A 4-mm ear biopsy was obtained from the rabbits (Miltex Instruments, York, PA). Birds were banded (US Fish and Wildlife Service, Patuxent, MD) and rabbits were ear-tagged (National Band and Tag, Newport, KY) prior to release. Wildlife research was approved by Michigan State University’s Institutional Animal Use and Care Committee, permit 02-07-13-000 and federal bird banding permit to RK.

### *Borrelia* species detection and nucleotide sequencing

Ticks were identified to species and stage using published keys [27,38]. Total DNA was extracted using the DNeasy Blood and Tissue Kit (Qiagen, Valencia, CA) as described in Hamer *et al.*[16]. Ear biopsies and nymphs were extracted individually, and conspecific larvae from the same individual animal were pooled for extraction. To confirm morphological identification, a subset of ticks were subjected to PCR and sequencing of the 5.8S rRNA – 28S rRNA gene internally transcribed spacer (ITS-2) [39]. The *B. burgdorferi* strain B31-infected nymphal *I. scapularis* acquired from the Centers for Disease Control and Prevention served as the positive controls. *Borrelia* spp. were detected using a nested polymerase chain reaction (PCR) for the 16S–23S rRNA intergenic spacer region (IGS) of *Borrelia* spp. [40]. PCR fragment sizes are approximately 1000 bp for *B. andersonii* and *B. burgdorferi*, and 500 bp for *B. miyamotoi*. Amplicons from positive reactions were purified (Qiagen, Valencia, CA) and subjected to direct DNA sequencing. Sequences were determined in both directions using an ABI Prism 3100 Genetic Analyzer (Applied Biosystems, Foster City, CA), and were compared to published sequences using the basic local alignment search tool in GenBank [41].

### Statistics

Chi-squared tests for independence were used to assess frequency of coinfestations. Logistic regression was used to assess tick infection over time. Minimum infection prevalence (MIP; i.e., assuming one positive larva per positive pool) was used for tests conducted on pooled larvae. The evolutionary history among strains within a *Borrelia* species was inferred using a neighbor-joining method in Mega5 in which the evolutionary distances were computed using the maximum composite likelihood method and are in the units of the number of base substitutions per site [42]. Novel strain sequences were deposited [GenBank:HMO15226-HMO152237 for *B. andersonii;* GU993309 for *B. miyamotoi*. The effect of sample size on strain richness was assessed using a web-based rarefaction calculator (University of Alberta, Edmonton, Canada; available at http://www.biology.ualberta.ca/jbrzusto/rarefact.php). Strain richness was estimated by using the nonparametric model of Chao-1, which considers the number of operational taxonomic units observed, and the frequency with which each was observed, to estimate total population strain richness [43].

## Results

A total of 12,301 ticks was removed from 19,631 bird captures (10.6% infestation prevalence) of which 86.4% were *I. dentatus,* 13.4% were *Haemaphysalis leporispalustris,* and <1% were *I. scapularis* and *Dermacentor variabilis.* Among the 105 avian species investigated, the bird species most commonly parasitized by ticks included Brown Thrasher (*Toxostoma rufum*), Lincoln’s Sparrow (*Melospiza lincolnii*), White-throated Sparrow (*Zonotrichia albicollis*), Eastern Towhee (*Pipilo erythrophthalmus*), Eastern White-crowned Sparrow *(Zonotrichia l. leucophrys),* Carolina Wren (*Thryothorus ludovicianus*), Song Sparrow (*Melospiza melodia*), Hermit Thrush (*Catharus guttatus*), American Robin (*Turdus migratorius*) and Fox Sparrow (*Passerella iliaca*) [36]. A total of 131 ticks was removed from 20 captures of eastern cottontails (75% infestation prevalence). The two most common tick species on cottontails were *I. dentatus* and *H. leporispalustris*, which parasitized 65 and 25% of captures, respectively. The PCR of the tick ITS-2 region resulted in molecular confirmation of the morphological identification for the subset of ticks that were tested (n = 17 which included representatives of all four tick species found on birds and rabbits).

### Phenology of bird-associated ticks

**Figure 2:**
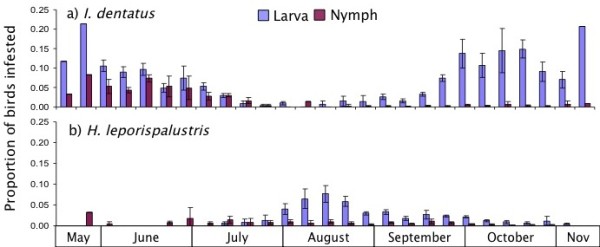
**Phenology of larval and nymphal bird-associated ticks.A**) *Ixodes dentatus*; **B**) *H. leporispalustris* depicted as weekly mean proportions of infested birds (error bars are standard error of the mean across years 2004–2007).

*I. dentatus* larvae exhibited bimodal peaks of attachment, with the earlier peak in June and the second peak in October-November, with both peaks of similar magnitude. *I. dentatus* nymphs, however, were mostly active in May-July, with smaller numbers throughout the fall (Figure 2A). There were 116 birds that had simultaneous infestations of *I. dentatus* larvae and nymphs. This frequency of coinfestations occurred ten times more commonly than expected by chance (Χ^2^ = 145.6; df = 3; P < 0.001). *Haempaphysalis leporispalustris* larvae were most active in August-September, whereas nymphs had a low level of activity throughout the sampling period with no discernable seasonal peak (Figure 2B). Across the study, the frequency of simultaneous infestations of *H. leporispalustris* larvae and nymphs on individual birds (44 birds) occurred approximately 22 times more commonly than expected by chance (Χ^2^ = 709; df = 3; P < 0.001). The majority of *H. leporispalustris* on birds were attached in between the peaks of *I. dentatus* attachment (Figure 2).

### Detection and diversity of *B. miyamotoi*

**Figure 3:**
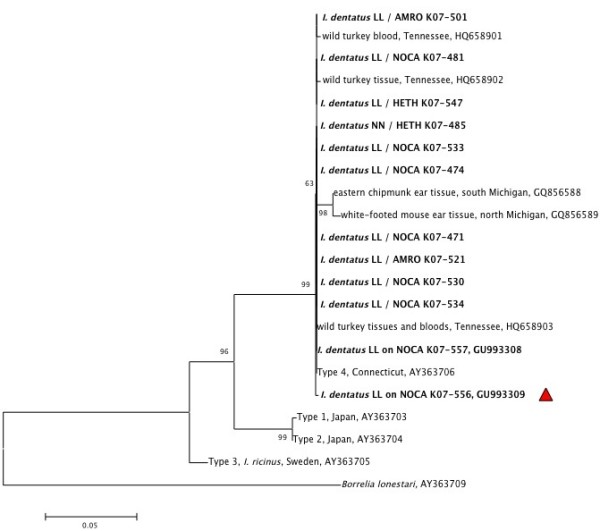
**Neighbor-joining phylogenetic tree based on 476 nucleotides of *B. miyamotoi *16S-23S rRNA IGS haplotypes collected from Pitsfield Banding Station, 2004–2007.** A sequence of *B. lonestari* obtained from GenBank was included as the outgroup. The twelve sequences generated in the current study are in bold and comprised two strain types, including 11 sequences that are identical to ‘Type 4’ (accession no. GU993308) and one novel strain indicated by the red triangle (GU993309). The percentages of replicate trees in which the associated taxa clustered together in the bootstrap test (1000 replicates) are shown next to the branches when 60 or higher. Sequences are labeled with the name of the tick species from which *B. miyamotoi* was amplified followed by the tick life stage/sex (NN = nymph; LL = larval pool), a four-letter alpha code indicating the avian host (AMRO = American Robin; HETH = Hermit Thrush; NOCA = Northern Cardinal), and a laboratory identification code. Additional sequences downloaded from GenBank comprise the reported genetic diversity of *B. miyamotoi* at the IGS locus and are labeled with spirochete origin.

All *B. miyamotoi*-positive samples were from bird ticks; none of 131 rabbit ticks or 20 rabbit ear tissues tested positive (Table 1). Regarding bird ticks, 15 of 2220 nymphs and larval pools (0.7%) were PCR-positive with sequences identical to or with significant sequence homology to *B. miyamotoi* (Table 1). There were an additional 22 samples that produced faint IGS bands at approximately 500 base pair size (suggestive of *B. miyamotoi*) that were not successfully sequenced. Accordingly, the reported infection prevalence should be considered a minimum. Fourteen of the 15 sequence-confirmed *B. miyamotoi*-positive samples were pools of larval *I. dentatus* and a single sample was a nymphal *I. dentatus*. All 15 *B. miyamotoi*-infected ticks were collected in 2007 (additional samples suspect-positive for *B. miyamotoi* were collected in earlier years, but were not successfully sequenced). *Borrelia miyamotoi* was only detected in samples from October and November, coinciding with peak *I. dentatus* larval phenology, with monthly prevalence of 1% and 5%, respectively. We removed *B. miyamotoi* -positive ticks/tick pools from hatch-year and after hatch-year individuals of three host species, including Northern Cardinal, American Robin, and Hermit Thrush. Whereas only 144 of the 1, 221 (11.8%) tick samples tested from October and November were derived from Northern Cardinals, this species contributed 11 of the 15 (73.3%) *B. miyamotoi*-positive samples; a frequency which is significantly higher than expected if infection were distributed evenly across species (Χ^2^ = 54.57; df = 1; p < 0.001). The two Hermit Thrush from which positive ticks were removed were fall migrants, whereas the Northern Cardinals and American Robins include individuals that were present during the summer breeding season as well as fall migrants. *Borrelia miyamotoi* IGS PCR products were successfully sequenced from 12 samples (including 11 larval pools and 1 nymph of *I. dentatus).* Of the 12 samples from which we obtained full IGS sequences, 11 were identical to the Type 4 published North American strain of *B. miyamotoi*[44], and a single sample from a larval *I. dentatus* pool had a single nucleotide polymorphism within the spacer (Figure 3).

**Table 1:** ***Borrelia *species infection prevalence in ticks and ear biopsies**

**Sample**		**N**	***B. burgdorferi *(%)**	***B. andersonii *(%)**	***B. miyamotoi *(%)**
Ticks from birds		2220	78 (3.5)	36 (1.6)	15 (0.7)
*H. leporispalustris*	larval pools	366	14 (3.8)	2 (0.5)	0
	nymphs	114	6 (5.3)	2 (1.8)	0
*D. variabilis*	larval pools	1	0	0	0
*I. dentatus*	larval pools	1467	44 (3.0)	6 (0.4)	14 (1.0)
	nymphs	263	13 (4.9)	24 (9.1)	1 (0.4)
	adult	1	0	0	0
*I. scapularis*	larval pools	2	0	1 (50)	0
	nymphs	6	1 (1.7)	0	0
Ticks from rabbits		131	4 (3.1)	7 (5.3)	0
*H. leporispalustris*	nymphs	2	0	0	0
	adults	7	0	0	0
*I. dentatus*	nymphs	2	0	2 (100)	0
	adults	120	4 (3.3)	5 (4.2)	0
Rabbit ear biopsies		20	4 (20)	1 (5)	0

Infection prevalence of three *Borrelia* spirochetes in ticks removed from birds and rabbits and rabbit ear biopsies collected in Michigan, 2004–2008. Sample sizes and infection prevalence are presented as both the sum across each sample type and specific to each tick species and life stage. The *B. burgdorferi* infection prevalences are taken from Hamer *et al*. [36]. Reported infection prevalences are considered a minimum due to a small number of additional samples with PCR bands indicative of a *Borrelia* species for which sequences were not obtained.

### Detection and diversity of *B. andersonii*

*Borrelia andersonii* infection was found in ticks removed from birds and rabbits and rabbit ear tissue. Regarding bird ticks, 35 of 2220 samples (1.6%) were PCR-positive with sequences identical to or with significant sequence homology to *B. andersonii* (Table 1)*;* positive samples included *I. dentatus* (n = 30 samples)*, H. leporispalustris* (4 samples), and *I. scapularis* (1 sample). Additionally, 18 samples were PCR-positive for *Borrelia* species with an IGS fragment at the expected size for *B. burgdorferi* or *B. andersonii*, but no sequences were obtained from these samples. As such, the reported tick infection prevalences are considered as a minimum.

The prevalence in nymphs was significantly greater than that of larval pools (6.2 and 0.5%, respectively; P < 0.0001). Positive samples were collected in all four years of the study, and annual variation in tick infection prevalence was not significant (R^2^ = 0.23; P = 1). Aggregating years, the highest monthly prevalence of 5.5 – 5.8% occurred in May and June, coinciding with peak *I. dentatus* nymphal phenology and the first larval activity peak, and prevalence significantly decreased over the season (R^2^ = 0.43; P < 0.0001). From mid-June through mid-August—a period that largely excludes the spring and fall migrations in our area—we detected 12 *B. andersonii*-positive ticks/pools, comprising 34.3% of all positives. Of these mid-summer positive samples, 10 (83.3%) were from hatch-year birds, indicative of local exposure. The 35 *B. andersonii*-positive samples were removed from 29 individual birds of 12 species. From the perspective of natural xenodiagnoses (i.e., identifying reservoir-competent host species based on production of infected larvae, in the absence of simultaneously attached infected nymphs), these results implicate six avian species as reservoir competent for *B. andersonii*: Brown Thrasher, Connecticut Warbler, Gray Catbird, Hermit Thrush, Swamp Sparrow, and Tufted Titmouse. Three individual birds were associated with more than one *B. andersonii*-positive sample collected either simultaneously or in a sequential capture.

Of the ticks removed from 20 rabbit captures, 5 of 120 (4.2%) adult and 2 of 2 (100%) nymphal *I. dentatus* were confirmed by sequencing as positive for *B. andersonii* (Table 1)*;* positive ticks came from 4 individual rabbits*.* None of the 7 adult or 2 nymphal *H. leporispalstris* tested positive for *Borrelia* species. Of the 20 rabbit ear biopsies, 1 (5%) was confirmed by sequencing as positive for *B. andersonii* (Table 1)*.* Additionally, 4 *I. dentatus* samples and 2 rabbit ear biopsies were PCR-positive for *Borrelia* species with an IGS fragment at the expected size for *B. burgdorferi* or *B. andersonii* (~1000bp), but no sequences were obtained from these samples. As such, the reported tick infection prevalences are considered as a minimum.

**Figure 4:**
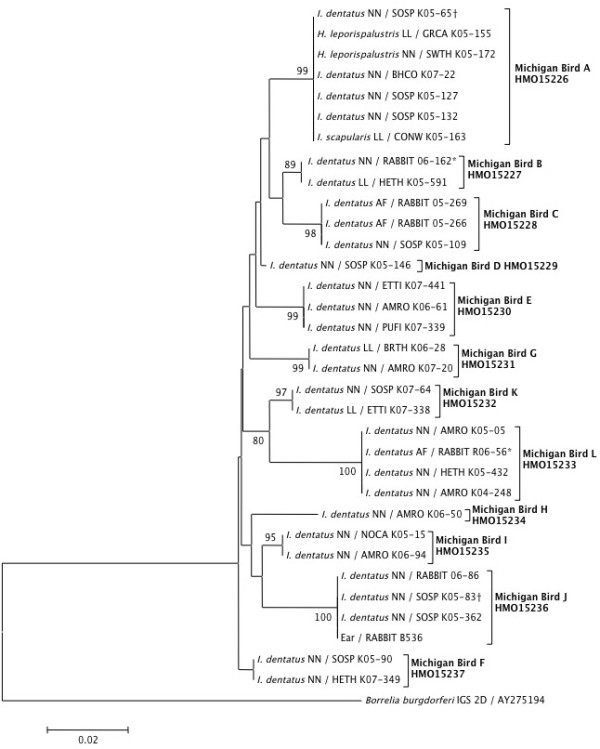
**Neighbor-joining phylogenetic tree based on 433 nucleotides of *B. andersonii *16S-23S rRNA IGS haplotypes collected from Pitsfield Banding Station, 2004–2007.** A sequence of *B. burgdorferi* obtained from GenBank was included as the outgroup. The percentages of replicate trees in which the associated taxa clustered together in the bootstrap test (1000 replicates) are shown next to the branches when 80 or higher. Sequences are labeled with the name of the tick species from which *B. andersonii* was amplified followed by the tick life stage/sex (NN = nymph; LL = larval pool; AF = adult female) and a four-letter alpha code indicating the avian host (AMRO = American Robin; BHCO = Brown-headed Cowbird; BRTH = Brown Thrasher; CONW = Connecticut Warbler; ETTI = Eastern Tufted Titmouse; GRCA = Gray Catbird; HETH = Hermit Thrush; NOCA = Northern Cardinal; PUFI = Purple Finch; SOSP = Song Sparrow; SWTH = Swainson’s Thrush) or RABBIT indicating the eastern cottontail, a laboratory identification code, followed by the GenBank accession number. The single sequence derived from rabbit ear tissue is labeled with ‘EAR’. The asterisk and dagger denote samples from the same individual host.

*Borrelia andersonii* IGS sequences from 33 samples (27 from larval pools and nymphs from birds, 5 from nymphs and adults from rabbits, and one from a rabbit ear) were subjected to phylogenetic analyses. We found 12 unique strains, 10 of which were represented by more than one sample in our population (Figure 4). One individual Song Sparrow and one individual rabbit each harbored two ticks infected with different strains of *B. andersonii.* Within the 493 nucleotide IGS fragment we analyzed, 57 sites were found to be polymorphic, including three indels (two single nucleotide indels, and one 10 nucleotide indel that was treated as a single polymorphism). Based on the observed strain richness and frequency, rarefaction analysis indicates that our detection of 12 strains encompasses most of the true strain richness, as the slope of the regression of number of strains versus sample size plateaus at a sample size of approximately 25. The Chao-1 non-parametric estimator of true species richness is 12.4 ± 0.72 strains.

## Discussion

Drawing upon current understanding of the ecology of *B. burgdorferi* and *B. miyamotoi*, within the context of *I. scapularis* phenologies in the eastern U.S., we can extrapolate cautiously to interpret and better understand the results found here regarding *B. andersonii* and *B. miyamotoi* infections in *I. dentatus* ticks attached to passerine birds in the Upper Midwestern U.S. Transovarial transmission for *B. burgdorferi* is not known to occur in *I. scapularis*[45], and thus, larvae hatch uninfected. Larvae and nymphs become infected horizontally by feeding on infected hosts, resulting in infected nymphs and adults [46]. The infection prevalence in questing adults is usually double that of questing nymphs in a given area and in part is attributed to each tick having two opportunities of becoming infected [47]. If *B. andersonii* is similar to *B. burgdorferi* in that transovarial transmission does not occur, then through implementation of natural xenodiagnosis our study implicates six avian species as competent reservoirs for *B. andersonii*; the presumption is that infected larval ticks obtained their spirochete infections from the birds upon which they were feeding at the time the sample was taken. *B. miyamotoi*, on the other hand, is transovarially transmitted in *I. scapularis*[14], and little is known about the contribution of any reservoir species to its persistence via horizontal transmission. Thus, in addition to nymphs and adult ticks being infected, questing larvae can be infected as well. Little data exist on the infection prevalence of questing larvae, but in general, the infection prevalences of questing nymphs and adults are low and are similar [12,18], suggesting that very little horizontal transmission occurs. While the reservoir competency of birds for *B. miyamotoi* using our study data cannot be established due to the potential for transovarial transmission, we suggest that *I. dentatus* tick and Northern Cardinals in particular may be important in the life history of *B. miyamotoi* due to the significant proportion of infected ticks contributed by this host species relative to its role in feeding ticks.

Tick phenology has implications for *Borrelia* species maintenance and infection dynamics of hosts. In eastern U.S. peak nymphal activity occurs in late spring/early summer [48]. In the Northeast, there is a small peak of larval activity in late spring/early summer, but the main peak is in late summer [49-51]. This asynchronous sequence of nymphs host-seeking prior to larvae allows for hosts to become infected by nymphs, and then to serve as a source of infection to larvae later in the season [48] and is believed to be a driving force in the high nymphal infection prevalence. Given the different modes of transmission between pathogens, in the Northeast peak *B. burgdorferi* transmission to hosts occurs in early summer, correlating with nymphal activity period, whereas peak *B. miyamotoi* transmission occurs in late summer, correlating with peak larval activity period [12]. The tick phenology is different in the northern Midwest, however, where the late spring/early summer larval activity period is often the main peak, coincident with the nymphal activity peak [37,48]. The more synchronous activity periods of nymphs and larvae is believed to results in some larvae feeding on hosts that have not yet been infected by nymphs, and therefore may contribute to lower nymphal infection prevalence in the Midwest compared to the Northeast. The synchronous phenology in the Midwest, however, may provide increased opportunities for co-feeding transmission, but presently its contribution is unknown.

In our study, we found that larval *I. dentatus* exhibited bimodal activity peaks in late spring/early summer and again in the fall, with nymphs active in the spring though mid-summer, similar to previous reports [52-54]. In the same way that *I. scapularis* nymphal activity prior to larval activity supports *B. burgdorferi* maintenance, the phenology we detected for *I. dentatus* is likely to support not only the low-level *B. andersonii* infection we report herein, but also our earlier findings of *B. burgdorferi* in the same ticks [36]. However, because larval activity was bimodal with the first larval peak coincident with nymphal activity, pathogen prevalence may be reduced relative to what would be expected if the activity of the two life stages were completely asynchronous. As a potential to further augment pathogen prevalence in ticks, we suggest the concept of co-feeding transmission in this system requires further attention, since significant frequencies of simultaneous co-infestations of the same avian hosts with both nymphs and larvae occurred. For example, we detected a Song Sparrow that simultaneously harbored *B. andersonii*-infected nymphs and larvae. The infection prevalence of *I. dentatus* adults attached to rabbits interestingly was lower/not significantly different from the infection prevalence of nymphs attached to birds; this contradicts the increasing pattern of *B. burgdorferi* infection in nymphs and adults of *I. scapularis*. This may suggest that *B. andersonii* transmission dynamics to *I. dentatus* nymphs differs from that of *B. burgdorferi* transmission dynamics to *I. scapularis* nymphs*.* Alternatively, the actual infection prevalence of *B. andersonii* in adult *I. dentatus* may have been higher, as there were four other *Borrelia*-positive ticks for which we do not know the species identity.

In contrast, the seasonal activity of nymphs relative to larvae may be less important for the maintenance of *B. miyamotoi* given the likely occurrence of transovarial transmission in *I. dentatus*. In addition to *I. scapularis*, *B. miyamotoi* has been found to be transovarially transmitted in *I. pacificus, I. ricinus, and I. persulcatus*. A majority of *B. miyamotoi*-infected ticks in our study were larvae removed from birds in the fall, a pattern that parallels the finding in which *B. miyamotoi* infection in mouse hosts rose toward the end of the summer coincident with the larval phenology [12]. A majority of *B. miyamotoi*-infected ticks occurred on Northern Cardinals, which are permanent residents in Michigan that typically stay within 8 km of where they were hatched [55], thereby demonstrating local acquisition of the spirochete. The presence of *B. miyamotoi*-infected ticks on two Hermit Thrushes, which do not breed in the area [56], affords a mechanism for migratory importation of the spirochete. Interestingly, we did not find any adult *I. dentatus* infected with *B. miyamotoi*, but given the low infection prevalence found in nymphs (0.7%) and the number of *I. dentatus* found on rabbits (n=120), there might not have been enough power to detect an infected tick.

The phenologies of *I. dentatus* and *H. leporispalustris* were strikingly opposite; this pattern is not explained by associations of the tick species with spring and fall migrants versus local/breeding birds, respectively. We therefore used *H. leporispalustris* as a bioassay or sentinel tick species to increase our ability to detect *Borrelia* spp. among birds across a broader temporal period and in a greater number of samples than would have been afforded by assessment of only *I. dentatus*, a known *Borrelia* spp. vector. Indeed four of the 480 *H. leporispalustris* pools removed from birds were infected with *B. andersonii*, though the ability of *H. leporispalustris* to transmit these agents remains unknown.

A high level of genetic diversity of *B. andersonii* was present within birds, rabbits, and their ticks at this focal site, with twelve unique IGS strains present within 33 sequenced samples derived from ticks removed from 11 host species. This strain richness, when standardized by sample size, is similar to what we reported previously for *B. burgdorferi* in the same samples (25 IGS strains present within 72 sequenced samples) [36]. However, whereas the statistical analysis indicated that the sampling in this study captured most of the *B. andersonii* strains estimated to be present in the system, this was not true for *B. burgdorferi,* where strain richness was estimated to be an order of magnitude higher than what we detected. Furthermore, in comparing the amount of evolutionary distance that separates the *B. andersonii* strains to that which separates the *B. burgdorferi* found within the same samples, we found that the former is much greater (0.02 nucleotide substitutions per site) than the latter (0.005 nucleotide substitutions per site on aphylogram assuming the same model of evolution as above). These data suggest that the duration of establishment of *B. andersonii* may be greater than that of *B. burgdorferi.* In this study location, where *I. scapularis* is not endemic, differences in diversity between *B. burgdorferi* and *B. andersonii* is not likely explained by adaptation to another tick, but may relate to differences in host range and differences in contributions by migratory birds, transmission efficiency, or other unmeasured factors.

At the other end of the spectrum, all but one of the 12 bird-associated *B. miyamotoi* sequences were identical at the IGS locus to ‘Type 4’ that has been reported previously from *I. scapularis* nymphs in Connecticut [44] and from wild turkey (*Meleagris gallopavo*) tissue and blood in Tennessee [17]. The novel Michigan variant had a single nucleotide polymorphism from Type 4, and was different from two mammal-associated variants we previously reported from the ear tissue of a white-footed mouse (*Peromyscus leucopus*) and an eastern chipmunk (*Tamias striatus*) collected at different sites in Michigan [16] (Figure 3). This low diversity of *B. miyamotoi* agrees with what has been found previously in *I. scapularis, I. ricinus, I. pacificus, and I persulcatus* (for example [18,44,56]).

Comparison of our current results with our previous report [36] of a minimum of 3.5, 3.1, and 20% *B. burgdorferi* infection prevalence in these bird ticks, rabbit ticks, and rabbit ears, respectively, confirms that individual mammals and birds are exposed to more than one *Borrelia* species. For instance, we amplified *B. andersonii* from the ear tissue of a rabbit that concurrently harbored *B. burgdorferi-*infected *I. dentatus,* and rabbits infected with *B. burgdorferi* harbored *B. andersonii*-infected ticks. We captured at least three birds that concurrently harbored ticks infected with *B. andersonii* and *B. burgdorferi,* and one bird that concurrently harbored ticks infected with *B. miyamotoi* and *B. burgdorferi*. Co-infections of *I. scapularis* with *B. burgdorferi* and *B. miyamotoi* has been reported previously: Barbour *et al*. [12] found that the frequency of co-infections in 7,205 questing nymphs from across the northern U.S. was not different than expected; Ullmann *et al*. [22] detected no coinfection in 250 *I. scapularis* nymphs from New Jersey, and Tokarz *et al.*[57] found that all 7 *B. miyamotoi*-infected adult *I. scapularis* in a sample of 286 from New York were co-infected with *B. burgdorferi*. The ecological and epidemiologic significance of co-infections with two or more *Borrelia* species requires additional study.

## Conclusions

Wild birds and ticks that specialize on birds and rabbits are involved in the natural maintenance and transmission of *B. miyamotoi* and *B. andersonii,* two spirochetes of potential public health importance. Understanding the ecology of cryptic vector species is of public health importance because they may augment the pathogen load in the environment that is a source of infection to sympatric bridge vectors, and furthermore may extend the time that reservoirs are infected and thereby allow more opportunity for transmission to bridge vectors. Given continued range expansions of *I. scapularis* from many endemic foci [58,59], including near our study region in Michigan [16], it is likely that humans will be exposed to these cryptic microbes with unknown health consequences. There has been a linear increase in the number of published genospecies of the *B. burgdorferi sensu lato* complex with 1–3 new species described per year since 1992, and our understanding of the clinical significance of the various members of the complex is continuing to evolve [60]. Studies of natural spirochete hosts and vectors may provide key information for understanding emerging human risk, as has been the case in the recognition of *B. bissettii* as a causative agent of human disease in Europe [61]. The co-occurrence of three *Borrelia* species within a host community and vector population highlights the need for diagnostic assays that can differentiate among these species for research, medical surveillance, and treatment [62].

## Abbreviations

MIP: Minimum infection prevalence; rRNA: Ribosomal RNA; ITS-2: Internally transcribed spacer; IGS: Intergenic spacer region; PCR: Polymerase chain reaction.

## Competing interests

The authors declare no competing interests.

## Authors’ contributions

SAH, JLS, and RK carried out the field collections. SAH and JLS carried out the diagnostic protocols, molecular genetic studies, and sequence alignments. SAH, GJH, JIT, and EW conceived the study, and participated in its design and coordination and helped to draft the manuscript. All authors read and approved the final manuscript.
